# The feasibility of gene therapy in the treatment of head and neck cancer

**DOI:** 10.1186/1758-3284-1-3

**Published:** 2009-01-12

**Authors:** Emanuela Vattemi, Pier Paolo Claudio

**Affiliations:** 1 Department of Clinical and Experimental Medicine, Section of Medical Oncology, University of Verona, Piazzale Stefani 1, 37126, Verona, Italy; 2 Department of Biochemistry and Microbiology, Joan C. Edwards School of Medicine, Marshall University, Huntington, WV 25755, USA; 3 Department of Surgery, Joan C. Edwards School of Medicine, Marshall University, Huntington, WV 25755, USA

## Abstract

Standard approach to the treatment of head and neck cancer include surgery, chemotherapy, and radiation. More recently, dramatic increases in our knowledge of the molecular and genetic basis of cancer combined with advances in technology have resulted in novel molecular therapies for this disease. In particular, gene therapy, which involves the transfer of genetic material to cells to produce a therapeutic effect, has become a promising approach. Clinical trials concerning gene therapy strategies in head and neck cancer as well as combination of these strategies with chemotherapy and radiation therapy will be discussed.

## Introduction

The prognosis of patients with Squamous Cell Carcinoma of the Head and Neck (SCCHN) is poor. The National Cancer Institute (NCI) reported in its SEER Cancer Statistic Review that there are approximately 40,000 new cases of primary head and neck cancer in the United States each year. The incidence of this cancer has been gradually increasing over the past 20 years and it is now the fifth leading cause of cancer incidence and the sixth leading cause of cancer-related death in the world [1]. Patients with early stage disease are treated with surgery or radiation therapy. However, despite advances in surgical resection, radiation techniques and adjuvant treatment, survival in these patients has not improved significantly over the past 30 years and one third of these patients develop local and/or regional tumor recurrence following surgery. In patients with locoregional disease, significant strides have been made in achieving excellent local control. However, nearly half of patients who undergo treatment for locally advanced disease will experience local or distant relapse. When patients experience treatment failure with first-line therapy, median survival time approaches 3 to 4 months, even with treatment. Therefore new treatment options are desperately needed and gene therapy offers hope in this regard. The goal of gene therapy is to introduce new genetic material into cancer cells that will selectively kill cancer cells with no toxicity to the surrounding non-malignant cells. In HNSCC this is an attractive treatment for two reasons. First, tumors are often accessible for direct injection of genetic therapeutic agents. Second, locoregional failure remains the predominant pattern of failure and cause of death among patients with recurrent disease. Recently, clinical trials of gene therapy in SCCHN have been completed and many data suggest the possibility and feasibility of this approach together with more conventional modality treatment such as radiation therapy and chemotherapy.

Gene therapy involves the introduction of foreign DNA into somatic cells to produce a therapeutic effect [2]. A variety of vectors have been used to transfer genes into cells. Viral vectors remain the gene transfer vehicles of choice, with retroviral and adenoviral vectors constituting 25% of all viral vectors currently in use in clinical trials. Several strategies have been developed for cancer gene therapy including 1) Replacement of tumor suppressor gene function; 2) Blockage of dominant oncogene function; 3) Oncolytic virus therapy, which selectively kill tumor cells but not normal cells; 4) Genetic prodrug activation therapy; 5) Genetic immunomodulation. These approaches may converge and can often be used in combination to amplify potential therapeutic effects. This review presents an update on the clinical results obtained in the field of HNSCC cancer gene therapy.

### Advexin (INGN 201, Ad5CMV-p53)

Advexin (INGN 201, Ad5CMV-p53; Introgen Therapeutics, Inc.) is an adenovirus (type 5) in which the E1 region is replaced with the cDNA of the p53 gene and is driven by a cytomegalovirus (CMV) promoter [3]. Deletion of the E1 region of the parental Ad5 DNA renders Advexin a replication-defective virus and prevents the expression of adenoviral genes. Studies with repeated sequencing showed that Advexin does not undergo mutational changes, and it maintains wild-type *p53 *DNA throughout the manufacturing process. The p53 gene is located on chromosome 17p in humans and it encodes a 393 amino acid protein that is critical to tumor biology [4]. Inactivation of p53 signaling pathways can allow proliferation of damaged cells and result in tumor formation. Delivery of the wild type p53 gene to a cancer cell via a modified adenoviral vector induces expression of wild-type p53 protein and triggers growth arrest or apoptosis, causing tumor growth inhibition.

A phase I trial carried out at the MD Anderson Cancer Center, Texas, USA, in patients with advanced local or regional head and neck cancer that was unresectable was reported by Clayman [5,6]. Thirty-three patients were treated by multiple intratumoral injections of Advexin at a dose of 10/11 pfu three times a week (this made up one course). Patients with resectable tumors received one full course of treatment and two additional administrations followed, one during surgery and one 72 hours after surgery. Patients with unresectable tumors received a treatment every 4 weeks. The treatment regimen was well tolerated and no serious side effects were reported. Patients with resectable versus non-resectable disease were analyzed separately. Of the non-resectable arm, 2 out of 17 patients had major responses (11.8%), six showed stable disease up to 3.5 months and nine showed progressive disease. Of the resectable arm, 4 of 15 patients remained free of disease with a median follow up of 18 months, which was greater than that expected for recurrent resectable SCCHN disease. In particular, one patient had a pathologic complete response at the time of surgery and remained free of disease 26 months after the initial treatment. Another patient also had no evidence of disease at 24 months. Moreover, analysis of tumor biopsies showed expression of the p53 transgene and evidence of apoptosis was also detectable. In this well conducted study not only issues pertaining to safety but also the kinetic of the vector employed were addressed. Ad-p53 was detected in blood by PCR by 30 minutes after injection and gradually eliminated over the next 48 hours. Cytopathic effect assays performed in patients treated at the highest 2-dose levels showed that viable Adp53 was present in blood at the highest levels 30 minutes after intratumoral injections, decreased at a rate of 2–4 orders of magnitude by 90 minutes and further decreased to very low or undetectable titers by 24 hours to be completely eliminated by 48 hours after injections. Ad-p53 was detected also in the urine from some patients who received doses of 3 × 10^9 pfu or greater and was present in urine from all patients who received doses of 3 × 10^10 pfu or greater. Ad-p53 detection in urine started within one day of the beginning of p53 injections. Urine was free of Adp53 within 3–17 days of the last Ad-p53 injections. Ad-p53 was also detected in the sputum and/or saliva samples of 6 high-dose patients tested. As with urine samples, Ad-p53 was detected within one day of injection, was present for several days after the last injection of the virus and was cleared to background levels within 7-days. Although Ad-p53 was detected in blood, urine and sputum, no patients reported viremic symptoms and 2 health providers with the greater risk of exposure were tested. No elevation of neutralizing antibodies was observed in their serum, and neither serum nor urine contained infectious p53 particles or Ad-p53 DNA.

Two phase II trials (T-201 and T-202) using this vector (ING-201) have been concluded at many centers and early results in 170 patients receiving intratumoral injection of Ad-p53 over a variety of doses and schedules showed that treatment with ING-201 is safe and effective [7]. The patient characteristics were similar in both trials, but the dosage was 50-fold greater in the study T201 than in T202. Although response rates were similar, the median survival duration was higher (6.2 vs. 3.8 months) and the mortality rate over the first 150 days (40 vs. 60) was lower in the high-dose compared with the low-dose study. Patients were not re-injected with Advexin during follow-up and this may have contributed to the loss of effect after the first 150 days. On the basis of these results, the use of higher doses and multiple administrations of Advexin were recommended for future studies. Another interesting data emerged from such studies while responses occurred regardless of the endogenous p53 status of the tumor. In fact, objective responses were documented in tumors in which no p53 mutations were found.

There are two ongoing phase III trials to compare the safety, efficacy and overall survival of treatment with Advexin as monotherapy or in combination with chemotherapy in patients with head and neck cancer. In one of these trials (T301), patients with local or regional recurrent refractory SCCHN who have failed radiation therapy and chemotherapy with platinum-containing drugs or taxanes were randomized to either Advexin intratumoral or methotrexate intravenously. Patients were treated for a maximum of nine cycles (27-weeks). Survival was the primary end point of the study and predicted accrual to accomplish this endpoint was 240 patients. In the second study (T302), patients with local or regional recurrent SCCHN who have not been previously exposed to chemotherapy were randomized to receive a combination of intratumoral Advexin, cisplatin and 5-fluorouracil (5-FU) or standard care with cisplatin/5-FU. The primary end point was time to progression and predicted accrual to accomplish this endpoint was 288 patients.

### Gencidine

Gendicine is another different molecular entity that combines an Adenovirus type 5 vector with a p53 expression cassette, using the RSV promoter and BGH (A) tail [8].

In a Phase I trial using the adenoviral vector SCH-58500, 16 patients with HNSCC received escalated doses ranging from 7.5 × 10^9 PFU to 7.5 × 10^12 PFU. Toxicity was limited to grade 1–2 fever and injection pain. One patient achieved a partial remission (PR), which correlated with the induction of apoptosis and transgene expression [9].

Recently, the first randomized clinical trial of p53 gene therapy was reported. Ninety patients with SCCHN were randomly allocated to receive either intratumoral injections of Ad-p53 in combination with radiation therapy (70 Gy/8 weeks) or radiation therapy alone. Complete remission was seen in 64.7% of patients receiving Ad-p53 combined with radiation therapy compared with 20% of patients receiving radiation therapy alone, which was statistically highly significant [10]. This clinical trial formed the basis for approval in head and neck cancer of Ad-p53 by the China State Food and Drug Administration, thus making Ad-p53 the first gene therapy approved for humane use [11,12].

### Onyx (DL1520)

ONYX is a replication-conditional adenovirus that is defective in the early regulator protein E1B, which binds to and inactivates p53 to promote its own activation [13]. Cells containing an intact p53 pathway are thus predicted to inhibit replication of an E1B 55 kD-deficient virus. In contrast, p53-deficient cells, such as those of a tumor, would be expected to allow efficient viral replication and subsequent cell killing. However several groups demonstrated that ONYX-015 efficiently replicates in many tumor cells types with wild-type p53 [14,15]. This apparent contradiction was resolved through examinations of p14ARF, a tumor suppressor gene whose product functionally stabilizes p53 [16]. Loss of p14ARF was identified as a mechanism that allows ONYX-015 replication in tumor cells retaining wild-type p53 [17].

A dose-escalation phase I trial was carried out by Ganly [18]. A total of 22 patients with recurrent head and neck cancer participated in this clinical trial. ONYX injection was performed intratumorally. This study showed no serious toxicity by intratumoral injection up to a viral dose of 1 × 10^11 plaque forming units (pfu) and dose-limiting toxicity was not reached at the highest dose of 10(11) plaque-forming units. However, using conventional response criteria, no objective responses were observed.

A subsequent phase II trial showed enhanced efficacy when the virus was given by multiple daily injections in the same group of patients [19,20]. In this study, patients either received single daily dose (n = 30) or fractionated twice daily dosing of dl1520 (n = 10). 14% of the patients receiving a single daily dose of dl1520 achieved a partial or complete response versus 10% of the patients receiving fractionated daily doses, which was not significant in this small study. Interestingly, response and viral replication was correlated with p53 status by gene sequencing and immunohistochemistry, with 58% (7 of 12) of p53 mutant tumors showing regression compared with no response among the p53 wild-type tumors. Furthermore, necrosis was confined to treated tumor tissue with no damage to adjacent normal tissue. Viral spread was documented in tumor tissue 5–14 days after treatment, even in the presence of high neutralizing antibody titers.

The viral construct DL1520 has also been employed in chemoprevention as a mouthwash preparation for patients with oral leucoplachia and displasia [21]. 19 assessable patients with histological confirmed oral dysplasia had ONYX-015 administered as a mouthwash. Three different regimens were studied. In regimen 1, the virus was given as a mouthwash at a dose of 1 × 10^10 pfu daily for 5 days, with cycles repeated four times weekly for a maximum of 12 cycles. Two of 4 patients treated had histological resolution of the dysplasia after six cycles. This was, however, short-lived, and the disease recurred in both patients. Therefore, regimen 2 employed a more frequent administration of virus at 1 × 10^10 pfu weekly for 24 weeks. In this regimen, four of twelve patients had complete resolution. This was, again, short-lived in two patients (recurrence at 24 weeks and 48 weeks, respectively), but a durable response was observed in one patient with no recurrence at a 30 months follow-up. In regimen 3, the virus was administered to three patients at a higher dose of 1 × 10^11 pfu daily for 5 days, followed by weekly administration for 5 weeks. In one patient, a complete response occurred that was also durable with no recurrence by 30 months post treatment. The treatment was well tolerated and histological resolution of displasia was seen in 37% of patients, although the effects were generally not sustained after discontinuation. This activity of ONYX-015 correlated with a decrease in p53 positivity. Nonetheless, this clinical trial establishes the need for a larger phase II/III trial to determine the activity of ONYX-015 mouthwash in the treatment of premalignant oral dysplasia. More detailed analysis of p53 status is also required, such as p53 gene sequencing, mdm2 expression, and p14ARF expression. In addition, combination treatment with novel agents that act in a p53-independent manner should also be considered. For example, isotretinoin works most effectively in dysplastic lesions without dysfunctional p53. Combination with ONYX-015 may therefore be additive due to the different mechanisms of action on tumor cells heterogeneous in p53 status. Moreover, potential synergy may exist between the two agents since isotretinoin has been shown to reverse differentiation in hyperplasic oral lesions. This would allow ONYX-015 to penetrate the basal layer of the epithelium of dysplastic lesions more effectively. Therefore, a phase II trial in combination with isotretinoin should also be considered.

Two interesting Phase II clinical trials have showed the feasibility and the preliminary efficacy of ONYX in association with chemotherapy [22,23]. Both these trials evaluated the use of intratumoral dl1520 injection in combination with cisplatin and 5-fluorouracil therapy in patients with recurrent squamous cell carcinoma of the head and neck. In the largest trial, reported by Khuri [22], 37 patients were enrolled and assessed for toxicity and 30 patients were assessable for response. Sixty-three percent of patients (19 of 30) had a measurable decrease in tumor size (> 50%). Eight of 30 (27%) had complete responses (no measurable disease), whereas 11 of 30 (36%) had partial regression (decrease of 50–100% in tumor area). Persistent response was confirmed 4 or more weeks after the initial response in 15 of the 19 patients (73%). After 6 months, none of the 19 tumors with an objective response had progressed, whereas all uninjected tumors treated with chemotherapy alone had progressed. In the smaller trial, reported by Lamont [23], 14 patients were enrolled and an overall response rate of 78% has been reported. These results are exciting if compared with historical data of a 14% measurable response (> 50%) in patients treated with dl1520 alone and 30–40% response for chemotherapy alone. The combination therapy was well tolerated and did not lead to an apparent increase in toxicity. The most frequent adverse event was injection-site pain (53%) that was mild (grade 1 or 2) and lasted less than 24-hours in the vast majority of affected patients. Only one patient discontinued therapy because of injection-site pain. Other grade 3 or 4 toxicity that occurred in patients receiving the combination included syncope, renal failure, facial edema, and anorexia. Most importantly, it was also shown that viral replication was not inhibited by this chemotherapy regimen. In addition, the response did not correlate with initial tumor size, presence of pretreatment neutralizing antibodies, TP53 gene status, or prior treatments. Median survival time was 10.5 months and the 1-year survival rate was estimated at 32%. Although the mechanism of the enhanced effects of combining oncolytic virotherapy and chemotherapy is unknown, several hypotheses exist. Intratumoral adenoviral replication results in the expression of proapoptotic molecules, including tumor necrosis factor, which presence could improve the efficacy of selected chemotherapeutic drugs in inducing apoptosis [24]. Another hypothesis rests on the fact that the induction of S phase by E1A adenoviral proteins could produce cell cycle-mediated chemo-sensitivity. Adenoviruses are known to induce the entry of cells into the S-phase, which could augment the effect of concurrently used chemotherapeutic agents. This hypothesis is further confirmed by the ability of adenoviruses to induce the expression of topoisomerase I [25]. Finally, E1A gene expression, occurring after ONYX-015 infection, can augment both p53-dependent and p53-independent tumor cell killing [26,27]. On the basis of these promising results an ongoing Phase III study, randomizing patients who have failed radiation therapy for recurrent head and neck cancer between treatment with ONYX-015 in combination with 5-FU/cisplatin versus 5FU/cisplatin alone is being conducted.

### H-101

H-101 is an E1B-55k and partly E3 deleted adenoviral vector comparable to ONYX-015. In a randomized phase III clinical trial of H-101 in combination with cisplatin and 5-FU, virus particles were administered by intratumoral injection daily for five consecutive days every three weeks [28]. A response rate of 39% was observed for chemotherapy alone, while chemotherapy and H-101 produced a 78% response rate. Common side effects were well tolerated by all the patients treated with H-101 an were fever, injection site reaction and influenza like symptoms. Survival data have not reported yet. On the basis of these results, in 2005 a Chinese company, Shanghai Sunway Biotech Co Ltd, was permitted to sell H-101 for the treatment of head and neck cancer in China.

### Liposome E1A (tgDCC-E1A)

The E1A gene functions as a tumor inhibitor by repressing oncoproteins and sensitizing cancer cells to chemotherapeutic and radiation treatments. The interaction of E1A, a nuclear phosphoprotein with a wide range of cellular proteins in multiple signal transduction pathways (cell cycle, DNA damage, histone deacetylation), results in multiple biological activities. E1A was initially appreciated for its ability to repress transcription, leading to down regulation of HER-2/neu protein and resulting in loss of malignant phenotypes. The anti-oncogenic activity of the E1A gene, however, is not limited to tumors that overexpress HER-2/neu. E1A also modulates expression of other genes, resulting in differentiation of certain cancer cells. It has also been shown to enhance antitumor activity in response to VP16, cisplatin, paclitaxel, and adriamycin. Several studies have also demonstrated a significant tumor radiosensitisation response to E1A therapy. Clinical investigations using cationic liposomes mixed with plasmid DNA encoding for E1A have shown safety and efficacy in animal models, as well as preliminary safety and activity in clinical trials. In one small pilot study, nine patients with recurrent, unresectable breast cancer and nine patients with recurrent, unresectable HNSCC, the E1A gene was administered using a lipid complex. No toxic effects were observed and the highest dose of drug treatment that does not cause unacceptable side effects (MTD) was not reached [29]. In a subsequent study, 24 patients with recurrent HNSCC have been treated with good tolerance [30]. However, clinical activity was modest with a median overall survival of only 4.6 months.

### HLA-B7 plasmid (Allovectin-7)

In 1 trial, 9 patients with advanced SCCHN who did not express the HLA-B7 antigen received intratumoral injection of an HLA-B7 plasmid (Allovectin-7). No toxic effects were observed. Four of 9 patients achieved a partial response and induction of HLA-B7 expression was confirmed in 2 of 4 patients who responded [31]. On the basis of these data an ongoing phase II trial involving multiple injections of Allovectin-7 have been started.

## Conclusions and future directions

The literature is full of preclinical studies describing gene therapy products employed as anticancer agents. However only a few of these approaches have been tested in clinical trials. This review focuses only on gene therapy strategies applied in clinical setting in head and neck cancer patients where the results of clinical trials have been reported.

Several important observations can be made from the clinical trials performed to date. Non-replicating and conditional replicating adenoviral vector are the most interesting gene therapy strategies explored in HNSCC. Intratumoral administration of these products is easy and possible, since these tumors are often accessible for direct injection.

As anticancer agents, viral products have an excellent profile and do not appear to enhance the toxicity of either chemotherapy or radiation making them good candidates for combined modality treatment strategies.

The most frequently reported adverse events with adenoviral products are fever and chill, asthenia, injection site pain, nausea and vomiting. However, the vast majority of these adverse events are mild to moderate.

Efficacy is limited to loco-regional control. In head and neck cancer, local and/or regional tumor recurrence develops in approximately one-third of patients, despite definitive treatment. Two-thirds of patients dying of this disease have no evidence of symptomatic distant metastases. Therefore, local and regional disease control is paramount in this disease, underscoring an urgent need for more effective local therapies. The promise of adenovirus-mediated gene therapy has not yet translated in patient survival primarily because of the inability to deliver the therapeutic gene to a large number of cells. Further optimization of vectors will be essential for the improvement of clinical effectiveness of cancer gene therapy.

Although preliminary clinical data are encouraging, well-designed studies are needed for evaluation of these new anticancer agents before their approval in clinical practice. Approval of adenoviral products in China for treatment of SCCHN has been subject of many controversies in the USA. Approval should be based on multi-center, multinational randomized studies planned to follow up patients in order to evaluate survival endpoint. Moreover, correlative endpoints, including assessment of expression of the transferred genes, immunologic response to transgenes and vectors, vector kinetics and verification of viral replication, if replicating vectors are employed, are crucial in order to validate the clinical utility of a given approach in combination with more traditional endpoint such as safety and efficacy.

Lastly, because gene therapy involves introducing changes to the body's set of genetic instructions, there are some important ethical issues that need to be considered on the use of gene therapy. Besides the classical concerns about the cost of gene therapy and availability to the general population, there are some safety issues that need to be considered. Recently, it has been described that somatic gene therapy resulted in an inheritable change to the genome of rats [32]. This phenomenon has been described as the breach of the Weismann barrier. The Weismann barrier is described as the principle that hereditary information flow from germline cells to the somatic cells only and not vice versa. The permeability of the Weismann barrier introduces new ethical problems (such those related to germline gene therapy) that were not considered previously when conducting somatic gene therapy and that will now need to be considered in the future.

## Competing interests

The authors declare that they have no competing interests.

## Authors' contributions

MV and PPC discussed the manuscript topics and drafted the manuscript.
